# Unintentional and self-poisoning mortalities in Mexico, 2000–2012

**DOI:** 10.1371/journal.pone.0181708

**Published:** 2017-07-20

**Authors:** Omar González-Santiago, Pilar C. Morales-San Claudio, Lucia G. Cantú-Cárdenas, Juan M. J. Favela-Hernández

**Affiliations:** Postgraduate Division, School of Chemical Sciences, Universidad Autónoma de Nuevo León, San Nicolás de los Garza, Nuevo León, México; Virginia University, UNITED STATES

## Abstract

**Introduction:**

Poisoning remains a major worldwide public health problem. Mortality varies by country, region and ethnicity. The objective of this study is to analyze recent trends in poisoning mortality in the Mexican population.

**Methods:**

Data regarding mortality induced by poisoning was obtained from a publicly available national database maintained by the National Institute of Statistics and Geography.

**Results:**

During the period from 2000 to 2012, average mortality rates for unintentional and self-poisoning were 1.09 and 0.41 per 100000 population, respectively. The highest mortality rate for unintentional poisoning was in older individuals of both genders while the highest mortality for self-poisoning was in older men and young women. Additional studies are needed in Mexico, especially those that analyze risk factors in older individuals and young women.

## Introduction

Poisoning remains a major worldwide public health problem and causes a considerable number of deaths. It accounts for approximately 1.2 million deaths and 1.7% of the total disease burden [1, 2]. Deaths from poisoning vary between regions and countries and by age, gender, ethnicity, substance involved, and intent [3, 4]. According to the World Health Organization (WHO), in 2012 an estimated of 193460 people died worldwide from unintentional poisoning (UP), 84% of these deaths occurred in low- and middle-income countries [5]. Drug prescriptions of mainly opioids, sedative, tranquilizers and antidepressants, are among the primary agents involved in poisoning deaths [2]. For example, in the United States opioid analgesics, benzodiazepines, and antidepressants accounted for the overwhelming increase in mortality and morbidity from drug overdose in the past two decades [6]. In contrast, the number of self-poisoning (SP) deaths has been difficult to estimate on a global level [7]; however, the WHO estimates that nearly a million people die each year due to suicide, with pesticides being the main cause (370000 deaths). Suicide accounted for 1.4% of all deaths worldwide in 2012 and 75% occurred in low- and middle-income countries [8].

Epidemiological studies of UP and SP deaths are scarce in Mexico. Reports exist regarding the non-fatal emergency visits due to poisoning in the pediatric population where the main cause has been drug prescriptions [9]. Environmental pollution, mainly with lead, is another source of poisoning in pediatric and adult population [10]. Considering the scarcity of studies regarding both non-fatal and fatal poisoning and since these studies are necessary to identify susceptible groups, we decided to analyze recent trends in poisoning mortality in Mexican population during 2000–2012

## Methods

Data were obtained from a publicly available national database maintained by the National Institute of Statistics and Geography (INEGI, in Spanish) [11]. The study period was chosen due to the availability of data since these records have a delay of 2–3 years. Deaths from UP, which include overdoses of illegal and legal drugs taken for nonmedical reasons, poisoning from legal drugs taken in error or at the wrong dose, and poisoning from other substances, were identified and collected according to the codes X40-X49 of the International Classification of Disease (ICD-10). SP deaths, which include deaths resulting from pesticides, gases or vapors, unspecified chemicals or noxious substances, alcohol, other drugs that act on the autonomic nervous system, narcotics, other sedatives; and hypnotics or antidepressants, were collected according to the ICD-10 codes X60-X69. The undetermined intent of poisoning was collected according to the ICD-10 codes Y10-Y19. In addition, an estimate of deaths from drug self-intoxication (DDSI) was calculated. DDSI included deaths due to drugs by suicide, accident or undetermined intent. We applied previously reported criteria; therefore the codes X40-X45 (unintentional), X60-X65 (intentional) and Y10-Y15 (undetermined) were selected for this purpose [12,13]. The UP and SP deaths were grouped by gender and six age groups (0–4, 5–9, 10–19, 20–39, 40–59 and ≥60 years). Due to the lack of SP deaths in children younger than 10 years, the first 3 groups were grouped into <19 years. To calculate the mortality rates in the years 2000, 2005 and 2010, we used the respective census data reported by the INEGI. The population for the remaining time was estimated by linear interpolation or extrapolation.

The adjusted mortality rates per 100,000 population were calculated for all groups with a direct method that used the World Standard Population [14]. A trend analysis was performed with a logarithmic regression of the natural logarithm of the adjusted rates and was tested with Student’s t-test, where the slope different from zero was the alternate hypothesis. The annual percent change (APC) was calculated with the following equation: APC = (e^m^–1)*100, where e raised to the m is the anti-ln of the slope of the logarithmic regression of rates. MINITAB 16 software was used for the analysis.

Because the data used in this study is in the public domain and easily accessible through the INEGI website [11], there was no need for approval from an ethics committee. Importantly, the database does not include any personal information regarding the deceased individuals.

## Results

During the study, a period a total of 21712 poisoning deaths occurred in México. Of these, 62.5% correspond to UP, 24.3% to SP and 13.2% to undetermined poisoning intent. According to gender and age, men and the 20–39-years age group presented the greatest number of poisoning deaths independent of intent (Table 1). With respect to main causative agents, gases were the main agent in women <19 years, and >60 years. Alcohol was the main agent in 40–59 years’ group and finally “Other and unspecified chemicals were the main agent in men and the 20–39-years group. The main causative agent according to intention was “other and unspecified chemicals” for UP while “pesticides” and “other and unspecified chemicals” was for SP and undetermined intent of poisoning, respectively; this pattern was independent of gender and age group. On the other hand, 6876 deaths were due to DDIS which represent 31.7% of all poisoning deaths.

**Table 1:** Number of deaths due to poisoning in Mexico (2000–2012).

		Unintentional	Self-poisoning	Undetermined	Total poisoning
Gender					
	Both	13573	5266	2873	21712
	Male	10267	2864	1941	15072
	Female	3306	2402	932	6640
Age					
	< 19 yrs	2287	987	482	3756
	20–39 yrs	5255	2425	1274	8954
	40–59 yrs	3607	1272	693	5572
	> 60 yrs	2424	582	424	3430

**Fig 1:**
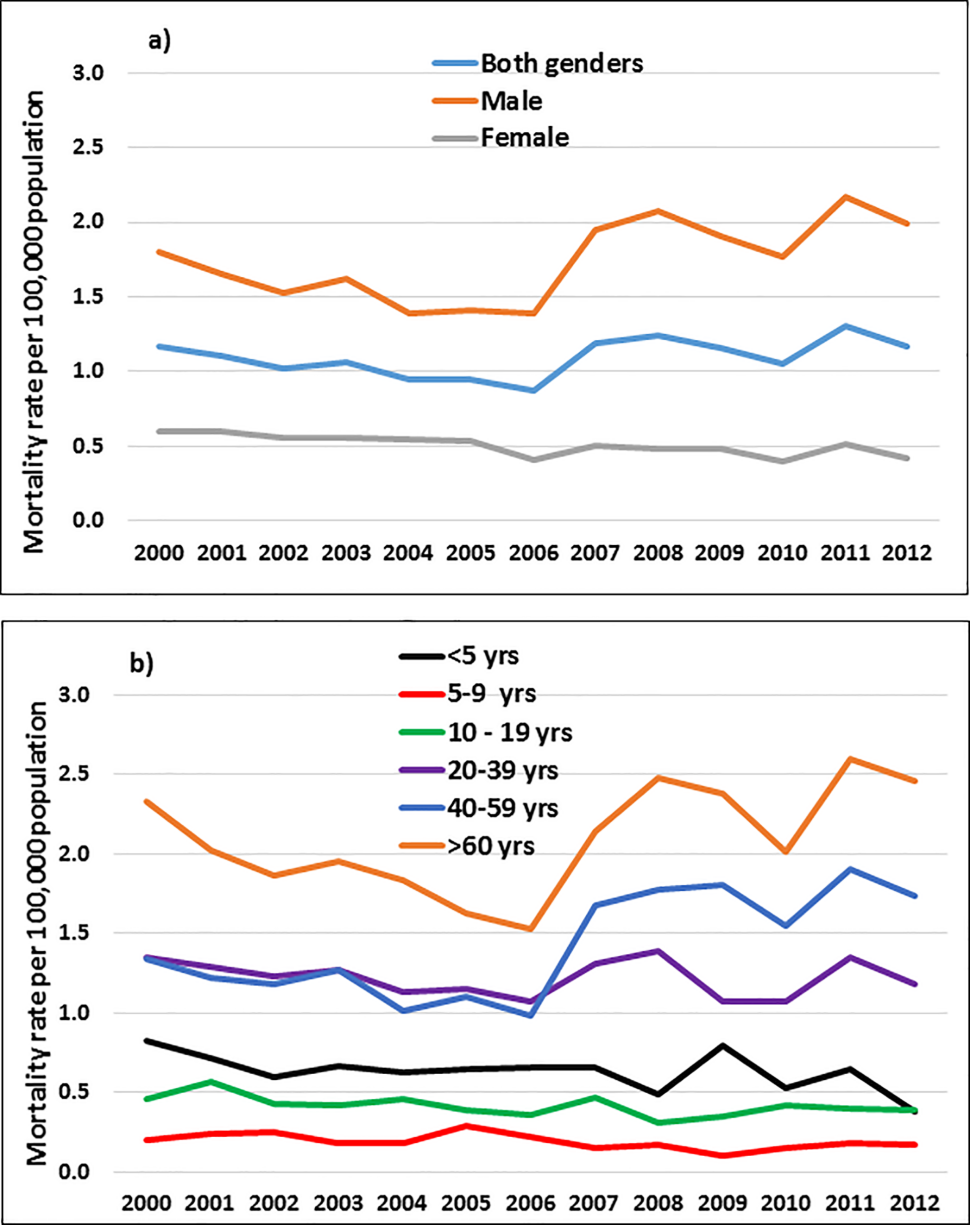
**Unintentional poisoning mortality in Mexico by a) gender and b) age group**.

The mean mortality rate of UP was 1.09 per 100,000 population. The death rate was higher for men than women (1.74 and 0.51 per 100,000 population, respectively) (Table 2). Moreover, the rate for men demonstrated a substantial increase during the study period from 1.79 to 1.99 per 100,000 population (APC = 2.36%) (Table 2). In contrast, the rate for women declined from 0.60 to 0.42 per 100,000 inhabitants, (APC = -2.73%) (Fig 1).

**Table 2:** Trends in unintentional and self-poisoning mortality in Mexico (rates are by 100000 population).

Type poisoning	Gender	Age (years)	2000	2012	Average	APC	P
**Unintentional**							
	Both genders	All ages	1,17	1,17	1,09	1,11	0,22
		<5	0,83	0,38	0,60	-3,05	0,04
		5–9	0,21	0,17	0,19	-3,87	0.04
		10–19	0,46	0,39	0,43	-2,18	**0,05**
		20–39	1,35	1,18	1,27	-0,61	0,41
		40–59	1,34	1,74	1,54	4,18	**0,01**
		>60	2,33	2,46	2,39	1,86	0,14
	Male	All ages	1,79	1,99	1,74	2,36	**0,04**
		<5	0,81	0,38	0,60	-3,15	**0,04**
		5–9	0,21	0,11	0,16	-7,69	**<0,01**
		< 19	0,56	0,58	0,57	-2,57	**0,22**
		20–39	2,33	2,04	2,19	-0,45	0,58
		40–59	2,33	3,23	2,78	5,49	**<0,01**
		>60	3,04	4,10	3,57	4,75	**<0,01**
	Female	All ages	0,60	0,42	0,51	-2,73	**<0,01**
		<5	0,84	0,37	0,61	-2,57	**0,20**
		5–9	0,20	0,23	0,21	0,38	**0,90**
		< 19	0,44	0,25	0,35	-2,37	0,07
		20–39	0,47	0,40	0,44	-1,54	0,08
		40–59	0,42	0,38	0,40	-2,14	0,12
		>60	1,70	1,01	1,36	-4,63	<0,01
**Self-poisoning**							
	Both genders	All ages	0,40	0,45	0,41	0,93	0,15
		< 19	0,19	0,20	0,18	0,09	0,95
		20–39	0,54	0,66	0,56	1,55	0,08
		40–59	0,49	0,51	0,51	0,66	0,40
		> 60	0,45	0,54	0,51	0,51	0,58
	Male	All ages	0,46	0,54	0,49	1,27	**0,02**
		< 19	0,11	0,13	0,10	2,10	0,18
		20–39	0,64	0,78	0,65	1,16	0,25
		40–59	0,58	0,67	0,69	1,51	0,16
		> 60	0,74	0,87	0,81	0,74	0,47
	Female	All ages	0,34	0,38	0,34	0,55	0,53
		< 19	0,26	0,28	0,25	-0,54	0,71
		20–39	0,45	0,56	0,47	2,03	**0,03**
		40–59	0,41	0,36	0,35	-0,70	0,62
** **		> 60	0,19	0,27	0,25	0,25	0,91

APC = Annual percentage change

Regarding age, an increase in rates was seen as age increased. In both genders, individuals ≥60 years of age had the highest mean mortality rate, whereas children, 5–9 years of age, had the lowest rate (2.39 and 0.19 per 100,000 population, respectively). This pattern of an increasing rate with age was observed in all age groups of both genders; moreover, men in the 40–59 years and ≤60 years’ groups presented a significant increase over time from 2.33 to 3.23 per 100,000 population (APC = 5.49%) and from 3.04 to 4.10 per 100,000 population (APC = 4.75%), respectively. Finally, women ≥60 years of age displayed a substantial decrease in mortality from 1.70 to 0.80 per 100,000 population (APC = -4.63%) (Table 2).

**Fig 2:**
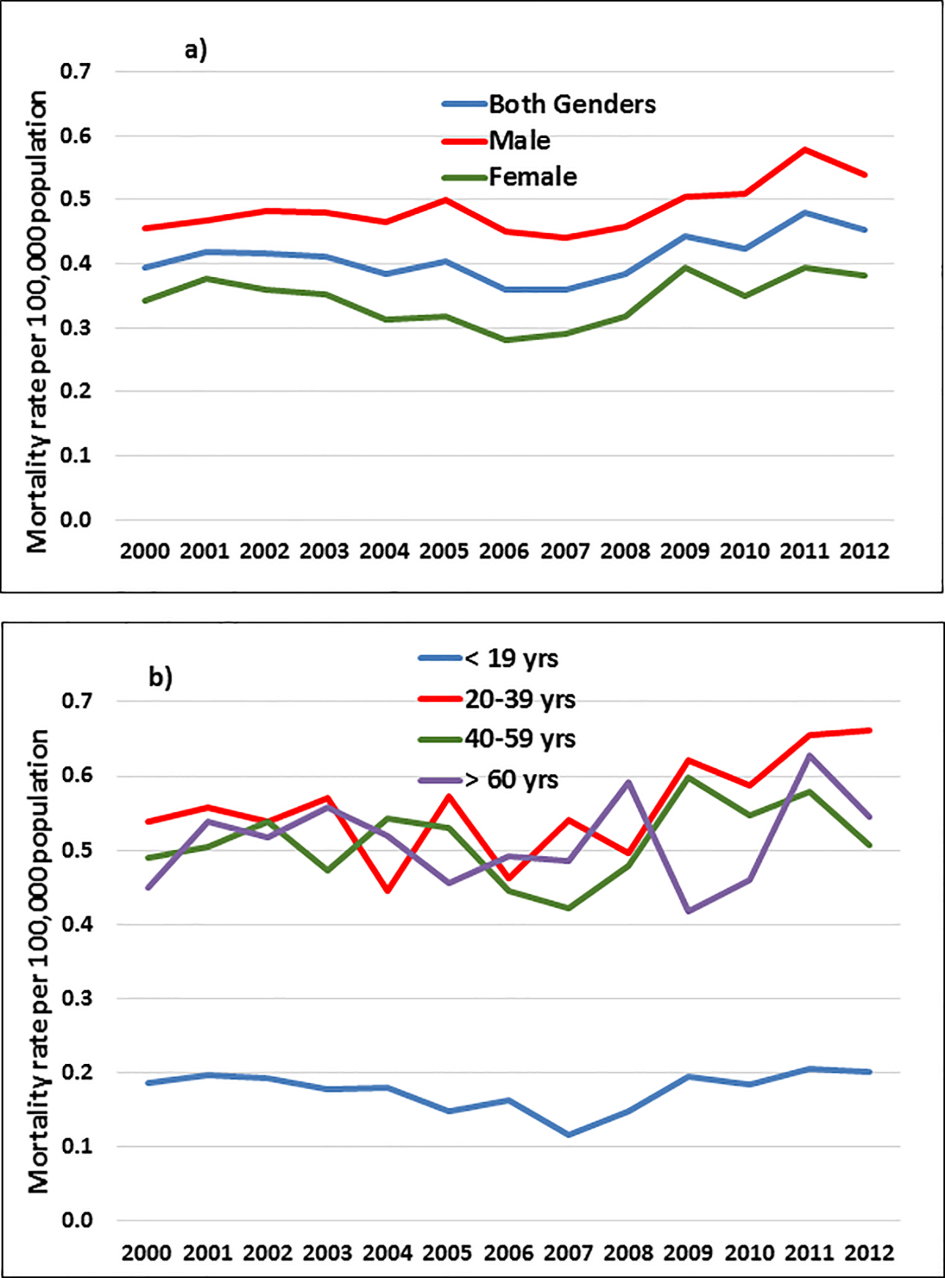
**Self-poisoning mortality in Mexico by a) gender and b) age group**.

The mean mortality rate for SP was 0.41 per 100,000 population. Men had higher mortality rates than women (0.49 and 0.34 per 100,000 population, respectively) and they presented a significant increase in mortality over time from 0.46 to 0.54 per 100,000 population (APC = 1.27%) (Fig 2). Individuals between 20–39 years and ≤19 years displayed the highest and lowest mortality rates for SP (0.56 and 0.18 per 100,000 population, respectively). In men, the highest mortality rate was observed in individuals ≥60 years of age, whereas the lowest mortality rate was observed in individuals ≤19 years of age (0.81 and 0.10 per 100,000 population, respectively). In women, the highest and lowest mortality rates were identified in the 20–39 and ≤19 years groups (0.47 and 0.25 per 100,000 population, respectively) (Table 2). In addition, women in the 20–39 years group had a significant increase in mortality from 0.45 to 0.56 per 100,000 population (APC = 2.03%).

## Discussion

In this study, we analyzed the mortality rates due to unintentional and self-poisoning in Mexico. Previous studies have analyzed total mortality by poisoning, but not specifically intentional or unintentional. Davalos *et al* in 2006 reported a decrease in all poisoning mortality rate from 2.0 to 1.5 per 100,000 population during 1981–2006 [15]. Compared with other developed countries, our results indicate that mortality rates for both types of poisonings are minor [16–20]. This could be partly explained by the distribution of population in Mexico. Compared with developed countries, Mexico has a predominantly young population. Another factor could be exactitude in the death register system. Total mortality rates of UP and SP do not show a significant trend during the studied period, although a decrease from 2000 to 2006 and an increase of 2007 to 2012 was observed. Reasons for this increase are not clear, but we speculate that could be due to a rise in pharmaceutical drug use and a greater availability of pesticides.

Mortality as a result of UP was higher in men than women. This result is consistent with other previous reports [16–20]. The reasons for this observation are not clear, but could be explained by men having more exposure to toxicants in work environments or easier access to poisons in general. Moreover, compared with women, men are more likely to smoke, drink and have an unhealthy diet; these factors could exacerbate the toxicity of poisons [19]. Our results differ from other studies that report higher mortality rates in middle age (approximately 40–60 years), independent of gender [16,17]. In our study, the highest mortality rate was identified in the oldest group (≥60 years), which is consistent with a previous report in Taiwan [18]. We speculate that loneliness and isolation of older people could influence these rates. It has been described that these factors are risk factors for mortality so it could apply also as a risk for UP [21, 22]. In addition, men and women ≥60 years of age presented significant trends in mortality over time. In the case of men, the rate underwent a significant increase, whereas the rate decreased for women. Other factor for high mortality in older Mexican individuals could be drug prescription availability. Polypharmacy, which is a risk factor for unintentional poisoning, is more prevalent in this age group [23]. Although children present low mortality rates, our results suggest that parents should enforce preventive strategies like keeping medicines or other substances out of their reach.

With respect to SP, men had higher mortality rates than women, which is consistent with other studies. This predominance in men could be explained by the greater effectiveness of the SP methods used [18, 23]. The different mortality peaks by age and gender are striking. In men, the peak occurred in individuals ≥60 years of age, whereas in women, the peak occurred at 20–39 years of age. This result is different from other reports that indicate higher mortality in middle-aged individuals [17, 23]. This inconsistency may be due to the fact that Mexican men ≥60 years of age are more predisposed than women to self-harm behavior in response to financial worries, social isolation, dependence, bereavement and physical disease. In contrast, the increased prevalence of substance abuse, mental illness and competition for jobs and resources could affect women in the 20–39 years group [24].

Our results emphasize the need to strengthen preventive strategies, especially in men ≥60 years of age and women between 20–39 years of age. One way to reduce both unintentional and self-poisoning mortalities is to limit access to illicit drugs, alcohol and medicinal drugs for individuals under psychological or emotional stress [25]. This requires efficient collaboration and communication between physicians and pharmacists, in addition to the implementation of drug monitoring programs by the Mexican Health Ministry (Secretaria de Salud, in Spanish).

Finally, we recommend interpreting our results with caution. It is important to consider that undetermined intent of poisoning is significant (13%) and could impact the mortality rates of both UP and SP. In addition, drug intoxication and other poisonings are among the methods most susceptible to misclassification and underreporting [12]. The high proportion of DDSI obtained in our study (31.7%) suggests that Mexico probably has a misclassification death counting problem. More studies are needed in this respect.

## Conclusions

Mortality by unintentional and self-poisoning is a considerable health problem in Mexico. Older individuals have the highest rates of unintentional poisoning. Young women and older men have the highest rates of self-poisoning mortality. More studies are necessary in México.

## Supporting information

S1 Table Raw data self poisoning Mexico. (XLS)

S2 Table Raw data unintentional poisoning Mexico. (XLS)

S3 Table Population Mexico 2000 2012. (XLS)
